# Supplementary material for: Peer Presence Effect on Numerosity and Phonological Comparisons in 4th Graders: When Working with a SchoolMate Makes Children More Adult-like
Source: Biology (Basel). 2021 Sep 12;10(9):902. doi: 10.3390/biology10090902 (PMC8470134; doi:10.3390/biology10090902)
Supplement: Supplementary file 1 [file biology-10-00902-s001.zip › biology-1295428-supplementary.pdf]

**Table S1: Individual estimated Ex-Gaussian parameters.** Ex-Gaussian decomposition of RTs into three parameters (μ, σ, τ) for each subject (Children or Adults) and each trial type (Numerosity or Phonological comparison).

| | Numerosity Comparison | | | Phonological Comparison | | |
|---|---|---|---|---|---|---|
| | μ | σ | τ | μ | σ | τ |
| Subject | Children – Alone Condition | | | | | |
| tt1 | 1.03 | 0.21 | 0.27 | 1.41 | 0.22 | 0.22 |
| tt10 | 1.1 | 0.15 | 0.2 | 1.49 | 0.26 | 0.2 |
| tt10RA | 1.44 | 0.29 | 0.14 | 1.57 | 0.33 | 0.17 |
| tt11 | 1.27 | 0.31 | 0.16 | 1.13 | 0.41 | 0.25 |
| tt12 | 1.05 | 0.26 | 0.2 | 1.44 | 0.22 | 0.17 |
| tt14 | 1.19 | 0.15 | 0.2 | 1.7 | 0.3 | 0.09 |
| tt15TL | 1.14 | 0.36 | 0.22 | 1.47 | 0.29 | 0.14 |
| tt16TC | 1.15 | 0.25 | 0.21 | 1.64 | 0.33 | 0.12 |
| tt18 | 1.11 | 0.38 | 0.17 | 1.51 | 0.38 | 0.13 |
| tt19GR | 1.05 | 0.16 | 0.21 | 1.37 | 0.24 | 0.13 |
| tt1BA | 1.04 | 0.37 | 0.22 | 1.02 | 0.25 | 0.25 |
| tt20GA | 0.86 | 0.27 | 0.15 | 1.51 | 0.28 | 0.16 |
| tt21 | 1.16 | 0.24 | 0.17 | 1.33 | 0.25 | 0.23 |
| tt22 | 0.63 | 0.2 | 0.26 | 1.1 | 0.2 | 0.14 |
| tt2CM | 0.66 | 0.18 | 0.12 | 0.79 | 0.15 | 0.25 |
| tt30SM | 0.95 | 0.4 | 0.21 | 1.49 | 0.36 | 0.15 |
| tt31SL | 0.87 | 0.17 | 0.2 | 1.01 | 0.17 | 0.26 |
| tt32MJ | 1.26 | 0.22 | 0.29 | 1.55 | 0.32 | 0.14 |
| tt33GA | 0.52 | 0.21 | 0.35 | 1.06 | 0.52 | 0.26 |
| tt34KJ | 1.12 | 0.21 | 0.15 | 1.39 | 0.31 | 0.23 |
| tt35JP | 1.36 | 0.24 | 0.15 | 1.42 | 0.27 | 0.17 |
| tt36GL | 1.12 | 0.27 | 0.17 | 1.12 | 0.22 | 0.22 |
| tt37FR | 0.92 | 0.31 | 0.21 | 1.1 | 0.33 | 0.26 |
| tt38CL | 1.33 | 0.29 | 0.16 | 1.17 | 0.3 | 0.4 |
| tt39BA | 1.05 | 0.3 | 0.25 | 1.24 | 0.31 | 0.21 |
| tt3NL | 0.73 | 0.09 | 0.09 | 0.92 | 0.15 | 0.25 |

| | | | | | | |
|---|---|---|---|---|---|---|
| tt40DO | 0.68 | 0.25 | 0.21 | 1.1 | 0.3 | 0.39 |
| tt41DA | 0.5 | 0.16 | 0.32 | 0.79 | 0.21 | 0.31 |
| tt42DL | 0.97 | 0.33 | 0.22 | 1.49 | 0.36 | 0.14 |
| tt4FD | 0.56 | 0.15 | 0.2 | 1.01 | 0.29 | 0.23 |
| tt6 | 0.58 | 0.15 | 0.25 | 1.11 | 0.34 | 0.3 |
| tt61NA | 1.41 | 0.18 | 0.24 | 1.39 | 0.21 | 0.28 |
| tt66MD | 0.74 | 0.18 | 0.26 | 1.07 | 0.2 | 0.26 |
| tt67DM | 0.34 | 0.12 | 0.22 | 0.51 | 0.17 | 0.34 |
| tt68BE | 1.21 | 0.23 | 0.31 | 1.48 | 0.22 | 0.29 |
| tt69AE | 0.87 | 0.23 | 0.3 | 1.5 | 0.34 | 0.17 |
| tt6GG | 1.23 | 0.14 | 0.22 | 1.49 | 0.24 | 0.17 |
| tt7 | 0.56 | 0.16 | 0.16 | 0.63 | 0.05 | 0.39 |
| tt74BE | 0.59 | 0.2 | 0.19 | 0.82 | 0.21 | 0.18 |
| tt75DN | 0.78 | 0.33 | 0.26 | 1.13 | 0.33 | 0.27 |
| tt76AE | - | - | - | 0.9 | 0.24 | 0.25 |
| tt77BM | 1.12 | 0.18 | 0.25 | 1.52 | 0.21 | 0.19 |
| tt8 | 1.26 | 0.23 | 0.13 | 1.3 | 0.21 | 0.23 |
| tt80VJ | 0.88 | 0.26 | 0.21 | 1.26 | 0.25 | 0.15 |
| tt81PG | 0.93 | 0.2 | 0.27 | 1.26 | 0.21 | 0.28 |
| tt82VN | 0.99 | 0.25 | 0.24 | 1.27 | 0.23 | 0.31 |
| tt85PF | 1.18 | 0.35 | 0.13 | 1.17 | 0.33 | 0.2 |
| tt9 | 0.8 | 0.19 | 0.31 | 1.15 | 0.31 | 0.14 |
| tt9MA | 1.18 | 0.27 | 0.2 | 1.53 | 0.25 | 0.2 |
| **Children – Social Condition** | | | | | | |
| tt11BI | 1.06 | 0.28 | 0.23 | 1.28 | 0.26 | 0.24 |
| tt12KC | 1.3 | 0.2 | 0.27 | 1.31 | 0.28 | 0.22 |
| tt13 | 0.85 | 0.24 | 0.33 | 1.79 | 0.15 | 0.2 |
| tt13FJ | 0.6 | 0.19 | 0.33 | 0.65 | 0.18 | 0.25 |
| tt14FC | 1.07 | 0.31 | 0.22 | 1.35 | 0.37 | 0.16 |
| tt15 | 1.1 | 0.19 | 0.25 | 1.5 | 0.37 | 0.17 |
| tt16 | 0.71 | 0.3 | 0.28 | 1.05 | 0.35 | 0.28 |

| | | | | | | |
|---|---|---|---|---|---|---|
| tt17 | 0.6 | 0.1 | 0.08 | 0.74 | 0.09 | 0.1 |
| tt19 | 1.04 | 0.34 | 0.11 | 1.13 | 0.3 | 0.3 |
| tt20 | 0.68 | 0.17 | 0.24 | 1.18 | 0.32 | 0.29 |
| tt21UH | 1.44 | 0.16 | 0.21 | 1.32 | 0.25 | 0.19 |
| tt22HC | 1.05 | 0.22 | 0.23 | 1.34 | 0.3 | 0.22 |
| tt23 | 0.33 | 0.07 | 0.15 | 0.57 | 0.14 | 0.07 |
| tt23MZ | 0.86 | 0.19 | 0.24 | 1.21 | 0.33 | 0.3 |
| tt24 | 0.58 | 0.18 | 0.3 | 1.42 | 0.42 | 0.13 |
| tt24TS | 0.64 | 0.1 | 0.28 | 0.96 | 0.18 | 0.29 |
| tt25 | 0.52 | 0.17 | 0.29 | 0.93 | 0.17 | 0.18 |
| tt26 | 1.3 | 0.29 | 0.14 | 1.55 | 0.23 | 0.05 |
| tt26MH | 0.51 | 0.19 | 0.2 | 1.1 | 0.33 | 0.23 |
| tt28NM | 0.29 | 0.03 | 0.2 | 0.74 | 0.2 | 0.16 |
| tt3 | 0.2 | 0.16 | 0.35 | 0.8 | 0.2 | 0.17 |
| tt4 | 0.31 | 0.15 | 0.34 | 0.58 | 0.08 | 0.11 |
| tt43BC | 0.48 | 0.03 | 0.32 | 0.93 | 0.2 | 0.25 |
| tt45LA | 0.51 | 0.22 | 0.37 | 1.26 | 0.33 | 0.19 |
| tt46SE | 0.43 | 0.18 | 0.3 | 0.64 | 0.12 | 0.16 |
| tt48LM | 0.5 | 0.09 | 0.22 | 0.79 | 0.14 | 0.33 |
| tt49DM | 1.07 | 0.17 | 0.23 | 1.14 | 0.24 | 0.23 |
| tt5 | 1.17 | 0.24 | 0.31 | 1.66 | 0.16 | 0.22 |
| tt50SA | 0.95 | 0.21 | 0.19 | 1.32 | 0.17 | 0.22 |
| tt53ED | 0.76 | 0.16 | 0.21 | 0.75 | 0.18 | 0.21 |
| tt54RA | 0.79 | 0.34 | 0.18 | 1.02 | 0.3 | 0.12 |
| tt55DC | 1.17 | 0.17 | 0.22 | 1.19 | 0.24 | 0.21 |
| tt56HM | 0.71 | 0.22 | 0.31 | 0.88 | 0.22 | 0.26 |
| tt57HA | 1.03 | 0.25 | 0.23 | 1.57 | 0.22 | 0.29 |
| tt58JR | 0.6 | 0.16 | 0.35 | 0.97 | 0.32 | 0.31 |
| tt59DN | 0.54 | 0.21 | 0.18 | 0.69 | 0.23 | 0.31 |
| tt60RG | 0.28 | 0.17 | 0.34 | 0.61 | 0.28 | 0.38 |
| tt62LL | 0.8 | 0.23 | 0.24 | 1.16 | 0.35 | 0.19 |
| tt63DT | 0.51 | 0.14 | 0.15 | 1.26 | 0.31 | 0.29 |

| | | | | | | |
|---|---|---|---|---|---|---|
| tt64GS | 0.51 | 0.16 | 0.31 | 1.17 | 0.45 | 0.18 |
| tt65JC | 0.56 | 0.14 | 0.19 | 1.03 | 0.29 | 0.09 |
| tt70BE | 0.76 | 0.18 | 0.24 | 1.15 | 0.25 | 0.24 |
| tt71MO | 0.52 | 0.11 | 0.22 | 1 | 0.15 | 0.14 |
| tt72BH | 0.89 | 0.23 | 0.22 | 1.43 | 0.25 | 0.33 |
| tt73CM | 0.41 | 0.13 | 0.26 | 0.77 | 0.12 | 0.12 |
| tt78IA | 1.06 | 0.32 | 0.26 | 1.59 | 0.36 | 0.14 |
| tt79DA | 0.78 | 0.31 | 0.31 | 1.35 | 0.4 | 0.14 |
| tt83PD | 0.97 | 0.21 | 0.18 | 1.64 | 0.17 | 0.23 |
| tt84SA | 0.44 | 0.17 | 0.18 | 0.68 | 0.17 | 0.13 |
| tt8BB | 1.17 | 0.34 | 0.14 | 1.12 | 0.28 | 0.21 |
| **Adults – Alone Condition** | | | | | | |
| tts10 | 0.99 | 0.06 | 0.08 | 1.07 | 0.04 | 0.14 |
| tts101 | 0.3 | 0.05 | 0.22 | 0.49 | 0.08 | 0.26 |
| tts102 | 0.41 | 0.16 | 0.29 | 0.68 | 0.24 | 0.26 |
| tts103 | 0.4 | 0.1 | 0.24 | 0.54 | 0.1 | 0.12 |
| tts106 | 1.2 | 0.15 | 0.21 | 1.47 | 0.24 | 0.08 |
| tts113 | 0.95 | 0.11 | 0.15 | 1.07 | 0.12 | 0.17 |
| tts114 | 1.23 | 0.18 | 0.09 | 1.22 | 0.15 | 0.14 |
| tts121 | 0.5 | 0.12 | 0.25 | 0.54 | 0.12 | 0.26 |
| tts122 | 0.83 | 0.23 | 0.18 | 0.65 | 0.19 | 0.23 |
| tts123 | 0.45 | 0.14 | 0.24 | 0.52 | 0.14 | 0.26 |
| tts124 | 0.31 | 0.07 | 0.17 | 0.48 | 0.07 | 0.19 |
| tts125 | 1.12 | 0.18 | 0.15 | 1.16 | 0.21 | 0.16 |
| tts126 | 0.71 | 0.16 | 0.22 | 0.69 | 0.16 | 0.28 |
| tts127 | 0.54 | 0.21 | 0.18 | 0.91 | 0.22 | 0.24 |
| tts128 | 1.1 | 0.15 | 0.17 | 1.21 | 0.18 | 0.17 |
| tts129 | 0.32 | 0.06 | 0.12 | 0.55 | 0.14 | 0.21 |
| tts132 | 0.39 | 0.12 | 0.15 | 0.44 | 0.05 | 0.23 |
| tts133 | 0.55 | 0.05 | 0.21 | 0.66 | 0.12 | 0.29 |
| tts134 | - | - | - | 1.35 | 0.14 | 0.19 |
| tts135 | 1.12 | 0.15 | 0.2 | 1.32 | 0.22 | 0.13 |

| | | | | | | |
|---|---|---|---|---|---|---|
| tts140 | 1.25 | 0.17 | 0.22 | 1.2 | 0.16 | 0.21 |
| tts145 | 0.41 | 0.06 | 0.22 | 0.38 | 0.11 | 0.2 |
| tts146 | 1.38 | 0.14 | 0.11 | 1.48 | 0.19 | 0.1 |
| tts147 | 0.98 | 0.23 | 0.19 | 1.15 | 0.2 | 0.16 |
| tts15 | 0.34 | 0.08 | 0.23 | 0.5 | 0.09 | 0.23 |
| tts18 | 0.97 | 0.3 | 0.16 | 1.14 | 0.28 | 0.16 |
| tts21 | 0.91 | 0.15 | 0.2 | 1.2 | 0.17 | 0.17 |
| tts22 | 0.45 | 0.12 | 0.17 | 0.4 | 0.11 | 0.24 |
| tts23 | 0.43 | 0.07 | 0.1 | 0.56 | 0.1 | 0.17 |
| tts24 | 0.73 | 0.24 | 0.23 | 0.7 | 0.2 | 0.28 |
| tts25 | 0.56 | 0.11 | 0.17 | 0.51 | 0.07 | 0.28 |
| tts26 | 1.05 | 0.13 | 0.15 | 1.05 | 0.07 | 0.17 |
| tts29 | 0.93 | 0.33 | 0.2 | 1.16 | 0.19 | 0.25 |
| tts3 | 0.41 | 0.03 | 0.19 | 0.6 | 0.11 | 0.2 |
| tts30 | 0.39 | 0.04 | 0.2 | 0.64 | 0.17 | 0.26 |
| tts31 | 1 | 0.06 | 0.17 | 1.06 | 0.06 | 0.11 |
| tts32 | 0.74 | 0.19 | 0.21 | 1.03 | 0.23 | 0.16 |
| tts33 | 0.85 | 0.15 | 0.2 | 1.01 | 0.22 | 0.15 |
| tts36 | 1.1 | 0.22 | 0.09 | 1.06 | 0.27 | 0.09 |
| tts37 | 0.28 | 0.12 | 0.21 | 0.5 | 0.06 | 0.21 |
| tts38 | 0.7 | 0.27 | 0.18 | 0.77 | 0.15 | 0.21 |
| tts41 | 0.33 | 0.05 | 0.22 | 0.47 | 0.07 | 0.11 |
| tts44 | 1.21 | 0.2 | 0.11 | 1.1 | 0.18 | 0.24 |
| tts45 | 0.95 | 0.13 | 0.17 | 1.14 | 0.15 | 0.13 |
| tts6 | 0.94 | 0.32 | 0.19 | 0.95 | 0.28 | 0.18 |
| tts7 | 0.37 | 0.07 | 0.16 | 0.52 | 0.07 | 0.2 |
| tts8 | 0.64 | 0.16 | 0.15 | 0.68 | 0.16 | 0.23 |
| tts9 | 1.15 | 0.24 | 0.06 | 1.08 | 0.17 | 0.22 |
| **Adults – Social Condition** | | | | | | |
| tts1 | 0.32 | 0.05 | 0.12 | 0.5 | 0.09 | 0.17 |
| tts104 | 0.33 | 0.06 | 0.12 | 0.43 | 0.02 | 0.16 |
| tts105 | 0.95 | 0.11 | 0.14 | 1.06 | 0.13 | 0.14 |

| | | | | | | |
|---|---|---|---|---|---|---|
| tts108 | 0.71 | 0.36 | 0.1 | 0.75 | 0.24 | 0.21 |
| tts109 | 1.02 | 0.15 | 0.1 | 0.96 | 0.12 | 0.16 |
| tts11 | 0.62 | 0.24 | 0.32 | 0.89 | 0.22 | 0.29 |
| tts110 | 1.32 | 0.18 | 0.03 | 1.38 | 0.19 | 0.07 |
| tts111 | 1.14 | 0.12 | 0.15 | 1.17 | 0.19 | 0.11 |
| tts112 | 0.61 | 0.17 | 0.19 | 0.73 | 0.17 | 0.22 |
| tts115 | 1.11 | 0.14 | 0.19 | 1.29 | 0.2 | 0.12 |
| tts116 | 1.19 | 0.1 | 0.14 | 1.22 | 0.13 | 0.17 |
| tts117 | 0.65 | 0.23 | 0.2 | 0.77 | 0.22 | 0.2 |
| tts119 | 0.37 | 0.11 | 0.19 | 0.7 | 0.21 | 0.21 |
| tts12 | 0.33 | 0.05 | 0.13 | 0.47 | 0.07 | 0.1 |
| tts120 | 0.49 | 0.13 | 0.13 | 0.47 | 0.02 | 0.2 |
| tts13 | 0.8 | 0.27 | 0.17 | 0.91 | 0.24 | 0.19 |
| tts130 | 0.54 | 0.21 | 0.22 | 0.94 | 0.33 | 0.19 |
| tts131 | 0.2 | 0.11 | 0.28 | 0.47 | 0.07 | 0.24 |
| tts136 | 0.41 | 0.12 | 0.12 | 0.67 | 0.16 | 0.17 |
| tts137 | 1 | 0.16 | 0.22 | 1.19 | 0.22 | 0.12 |
| tts138 | 0.54 | 0.16 | 0.24 | 0.76 | 0.16 | 0.2 |
| tts139 | 0.25 | 0.09 | 0.22 | 0.59 | 0.19 | 0.24 |
| tts14 | 0.51 | 0.18 | 0.21 | 0.69 | 0.2 | 0.24 |
| tts141 | 0.57 | 0.21 | 0.25 | 0.81 | 0.22 | 0.21 |
| tts142 | 0.89 | 0.27 | 0.1 | 0.84 | 0.27 | 0.19 |
| tts143 | 0.44 | 0.17 | 0.26 | 0.5 | 0.1 | 0.29 |
| tts144 | 0.73 | 0.23 | 0.15 | 0.79 | 0.21 | 0.12 |
| tts150 | 0.34 | 0.06 | 0.1 | 0.56 | 0.11 | 0.14 |
| tts152 | 1 | 0.12 | 0.16 | 1.19 | 0.1 | 0.13 |
| tts153 | 0.71 | 0.29 | 0.21 | 0.77 | 0.27 | 0.23 |
| tts154 | 0.44 | 0.12 | 0.16 | 0.53 | 0.1 | 0.17 |
| tts155 | 0.55 | 0.22 | 0.25 | 0.59 | 0.25 | 0.29 |
| tts16 | 1.04 | 0.17 | 0.11 | 1.17 | 0.21 | 0.14 |
| tts17 | 0.45 | 0.13 | 0.21 | 0.66 | 0.17 | 0.2 |
| tts19 | 0.46 | 0.13 | 0.15 | 0.59 | 0.13 | 0.15 |

| | | | | | | |
|---|---|---|---|---|---|---|
| tts2 | 0.18 | 0.1 | 0.25 | 0.38 | 0.07 | 0.26 |
| tts20 | 0.43 | 0.08 | 0.14 | 0.44 | 0.05 | 0.27 |
| tts27 | 0.68 | 0.16 | 0.15 | 0.6 | 0.09 | 0.23 |
| tts28 | 0.33 | 0.07 | 0.09 | 0.42 | 0.03 | 0.13 |
| tts34 | 0.91 | 0.09 | 0.12 | 0.99 | 0.13 | 0.22 |
| tts35 | 0.48 | 0.13 | 0.19 | 0.51 | 0.05 | 0.26 |
| tts39 | 0.28 | 0.1 | 0.2 | 0.51 | 0.11 | 0.16 |
| tts4 | 0.44 | 0.13 | 0.11 | 0.58 | 0.15 | 0.26 |
| tts40 | 0.31 | 0.05 | 0.2 | 0.48 | 0.11 | 0.15 |
| tts42 | 1.14 | 0.13 | 0.17 | 1.24 | 0.19 | 0.11 |
| tts43 | 0.77 | 0.25 | 0.15 | 1.27 | 0.16 | 0.21 |
| tts46 | 0.37 | 0.14 | 0.25 | 0.98 | 0.3 | 0.18 |
| tts47 | 0.62 | 0.19 | 0.25 | 1.13 | 0.27 | 0.14 |
| tts5 | 1.18 | 0.22 | 0.07 | 1.26 | 0.3 | 0.06 |

**Figure S1: Individual estimated Ex-Gaussian distributions.** Individual RT distributions (correct trials, black curve) in children (A) and adults (B) for each trial type (Numerosity or Phonological comparison) and each condition (Social/Alone) and estimated distribution according to the Ex-Gaussian model estimated parameters (red curve).

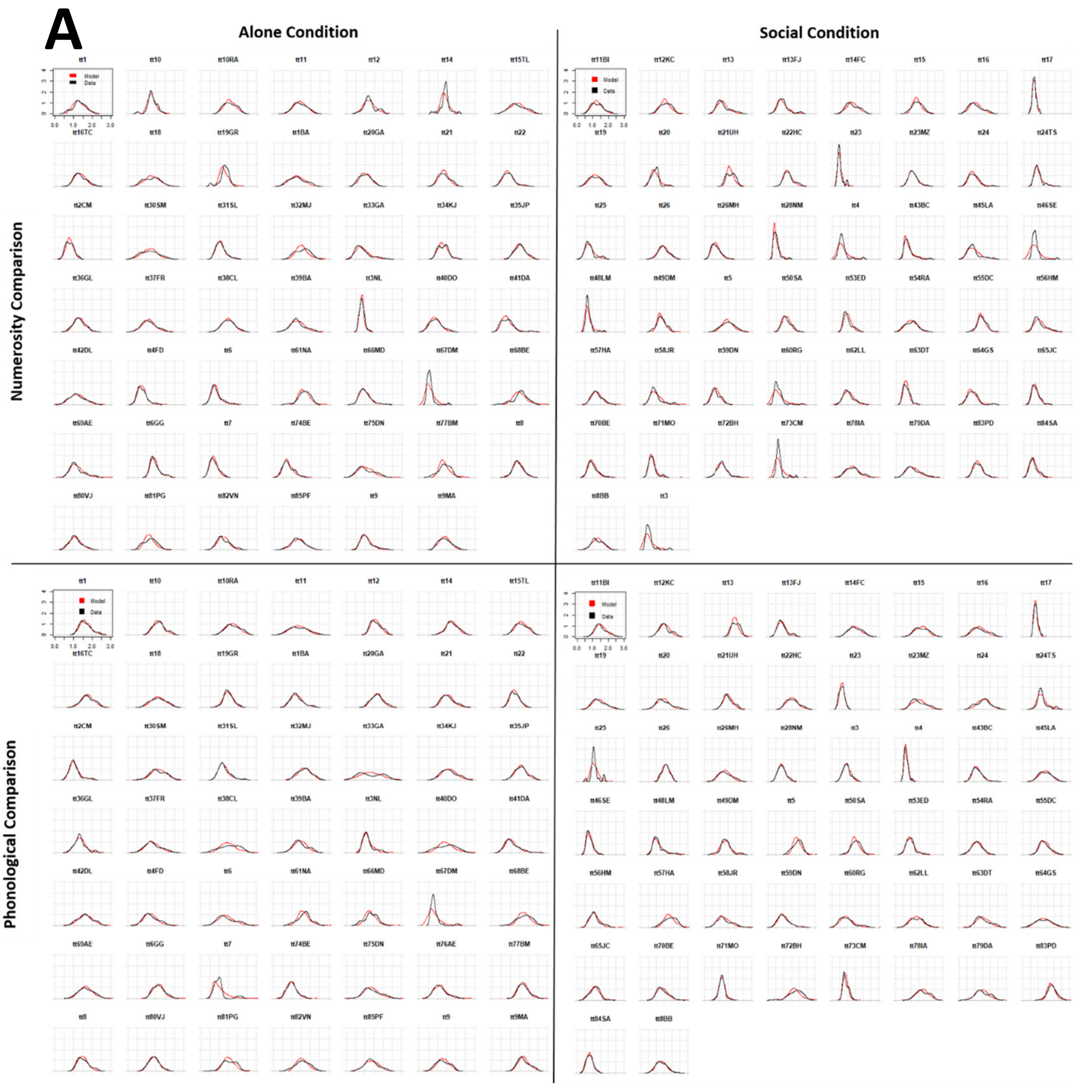

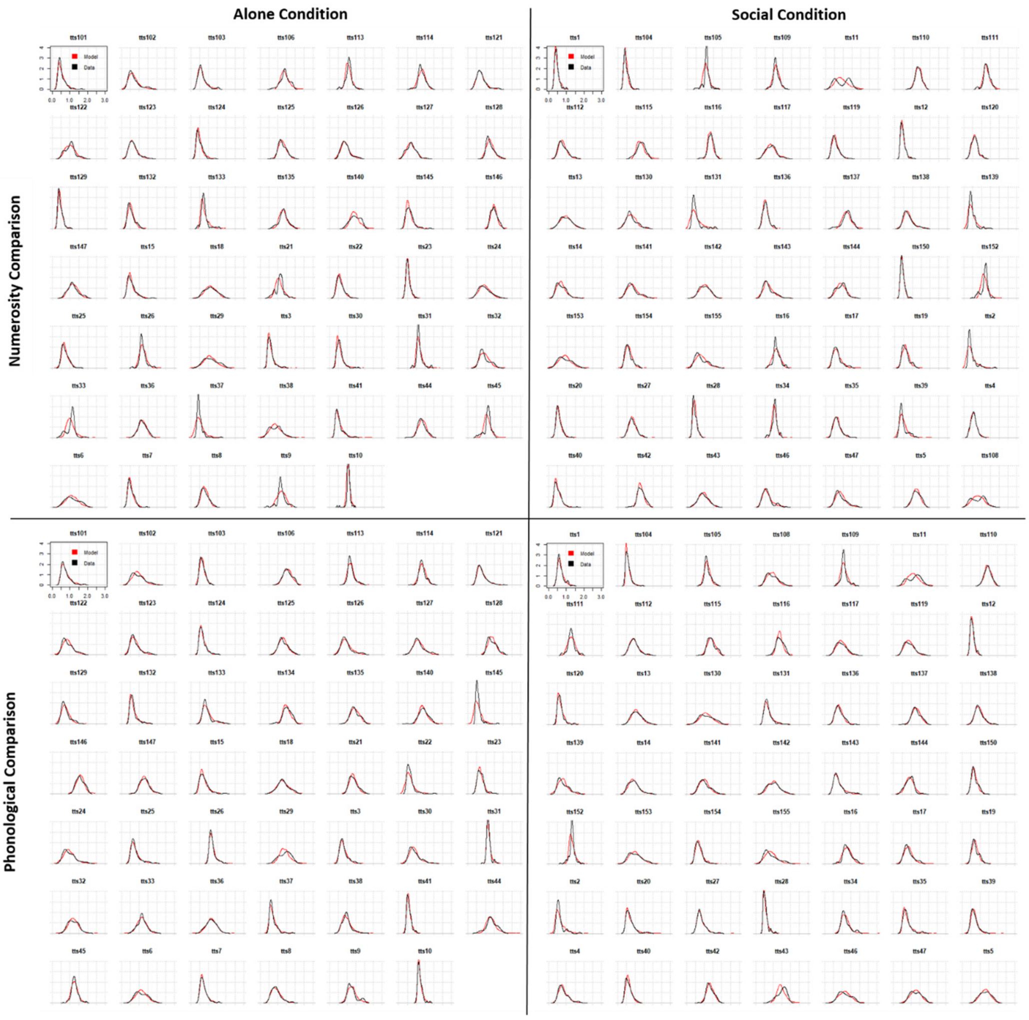
Alone Condition
Social Condition
Numerosity Comparison
Phonological Comparison
Model
Data

B

**Table S2: Individual estimated Diffusion model parameters.** Diffusion model estimated parameters (a, v, t0, szr, sv, st0) and fitting index for each subject (Children or Adults) and Trial type (Numerosity or Phonological comparison). A value of fitting index close to 1 indicates a good fitting.

| | Numerosity Comparison | | | | | | | Phonological Comparison | | | | | | |
|---|---|---|---|---|---|---|---|---|---|---|---|---|---|---|
| | a | v | t0 | szr | sv | st0 | Fitting index | a | v | t0 | szr | sv | st0 | Fitting index |
| Subject | Children – Alone Condition | | | | | | | | | | | | | |
| tt20GA | 1.5002 | 2.0866 | 0.6728 | 0.0002 | 0.0139 | 0.8530 | 0.9998 | 1.1539 | 1.0431 | 1.4235 | 0.5372 | 0.7915 | 0.7778 | 1.0000 |
| tt22 | 2.3118 | 3.0038 | 0.4790 | 0.1156 | 0.2975 | 0.6825 | 0.9987 | 1.6025 | 1.7887 | 0.8578 | 0.5428 | 0.2312 | 0.5821 | 0.9861 |
| tt2CM | | | | | | | | | | | | | | |
| tt31SL | 1.1449 | 1.2806 | 0.5561 | 0.4506 | 0.5901 | 0.4554 | 0.9883 | 1.2795 | 1.9456 | 0.7681 | 0.2557 | 0.8917 | 0.4367 | 0.9998 |
| tt33GA | 1.6088 | 2.5381 | 0.7418 | 0.1374 | 0.7722 | 0.5107 | 0.9857 | 1.2652 | 0.7725 | 0.9361 | 0.3338 | 0.4376 | 0.3177 | 0.9999 |
| tt37FR | 1.5644 | 0.7864 | 0.3623 | 0.4199 | 0.5000 | 0.2025 | 0.9998 | 1.9362 | 0.1953 | 0.5041 | 0.1274 | 0.1854 | 0.9959 | 0.7910 |
| tt3NL | | | | | | | | | | | | | | |
| tt40DO | 1.6121 | 1.2361 | 0.7022 | 0.4715 | 0.3264 | 0.5294 | 0.9997 | 1.5954 | 0.6773 | 0.8531 | 0.2062 | 0.5853 | 0.4847 | 0.9979 |
| tt41DA | 0.8719 | 1.9244 | 0.6900 | 0.0005 | 0.3282 | 0.2175 | 0.9956 | 1.1973 | 0.7116 | 0.8424 | 0.2905 | 0.4647 | 0.2560 | 0.9998 |
| tt42DL | 1.6523 | 1.2657 | 0.4057 | 0.1193 | 0.3271 | 0.6715 | 0.9892 | 1.5869 | 0.8225 | 1.0161 | 0.5469 | 0.5229 | 1.3986 | 0.9910 |
| tt4FD | 1.9134 | 1.9935 | 0.3503 | 0.3125 | 0.3705 | 0.6989 | 0.9355 | 1.7891 | 1.3874 | 0.5883 | 0.3046 | 0.5401 | 0.2372 | 0.9995 |
| tt6 | | | | | | | | | | | | | | |
| tt66MD | 1.6630 | 0.9493 | 0.7426 | 0.1073 | 0.6322 | 0.5298 | 0.9824 | 1.5208 | 0.8601 | 1.2543 | 0.4567 | 0.7820 | 0.8375 | 0.9923 |
| tt67DM | 1.3233 | 1.2845 | 0.4470 | 0.2404 | 0.6908 | 0.2074 | 0.9752 | 1.5525 | 0.6604 | 0.7422 | 0.0599 | 0.6596 | 0.4725 | 0.9998 |
| tt69AE | 1.2284 | 0.9499 | 0.5290 | 0.0957 | 0.7030 | 0.2757 | 0.9968 | 1.7967 | 0.8852 | 0.7978 | 0.3868 | 0.2386 | 0.3634 | 0.9996 |
| tt7 | 1.6823 | 1.9144 | 0.6030 | 0.2403 | 0.4396 | 0.4408 | 0.9980 | 1.3150 | 0.9831 | 1.0252 | 0.3113 | 0.0879 | 0.7366 | 0.8799 |
| tt74BE | | | | | | | | | | | | | | |
| tt75DN | 0.7744 | 1.6534 | 0.4174 | 0.2095 | 0.3642 | 0.2595 | 0.9998 | 1.0389 | 2.0337 | 0.6222 | 0.1292 | 1.2734 | 0.2191 | 0.9999 |
| tt80VJ | 1.7294 | 1.1464 | 0.6762 | 0.0779 | 0.6068 | 0.0580 | 0.9993 | 1.5231 | 0.3530 | 1.1523 | 0.3636 | 0.4537 | 0.8655 | 0.9997 |
| tt82VN | 1.2643 | 1.5067 | 0.4386 | 0.2597 | 0.5152 | 0.2209 | 0.9980 | 1.0916 | 1.2996 | 0.6620 | 0.2888 | 0.5711 | 0.6108 | 0.9184 |
| tt9 | | | | | | | | | | | | | | |

| | | | | | | | | | | | | | | |
|---|---|---|---|---|---|---|---|---|---|---|---|---|---|---|
| tt1<br>tt10<br>tt10RA<br>tt11<br>tt12<br>tt14<br>tt15TL<br>tt16TC<br>tt18<br>tt19GR<br>tt1BA<br>tt21<br>tt30SM<br>tt32MJ<br>tt34KJ<br>tt35JP<br>tt36GL<br>tt38CL<br>tt39BA<br>tt61NA<br>tt68BE<br>tt6GG<br>tt77BM<br>tt8<br>tt81PG<br>tt85PF<br>tt9MA<br>tt76AE | 1.5696 | 1.7481 | 0.3917 | 0.2703 | 0.4647 | 0.4986 | 0.9915 | 1.4667 | 0.9081 | 0.5866 | 0.4607 | 0.2654 | 0.3833 | 0.9602 |
| | 1.6191 | 0.1653 | 0.4089 | 0.3603 | 0.2386 | 0.4664 | 0.9991 | 1.7468 | 0.8093 | 0.8520 | 0.5227 | 0.4498 | 0.0552 | 0.9988 |
| | 1.6521 | 1.3581 | 0.6880 | 0.2126 | 0.4782 | 0.3894 | 0.9989 | 1.4374 | 2.1128 | 1.1302 | 0.0287 | 0.9053 | 0.6353 | 0.9999 |
| | 1.7097 | 1.2687 | 0.7664 | 0.3325 | 0.5401 | 0.2385 | 0.8788 | 1.4506 | 1.1045 | 1.2018 | 0.5341 | 0.0602 | 0.8705 | 0.9996 |
| | 1.5340 | 1.1703 | 0.6534 | 0.1170 | 0.6983 | 0.2925 | 1.0000 | 1.4531 | 0.3294 | 0.7938 | 0.1142 | 0.2637 | 0.7592 | 0.9958 |
| | 1.6180 | 1.3795 | 0.9266 | 0.1082 | 0.3966 | 0.3429 | 0.9771 | 1.6057 | 1.3248 | 1.2206 | 0.5091 | 0.3140 | 0.2420 | 1.0000 |
| | 1.2386 | 1.3845 | 1.0709 | 0.1295 | 1.2432 | 0.2432 | 0.9999 | 1.5212 | 0.7018 | 1.2932 | 0.0001 | 0.8393 | 0.2485 | 0.9923 |
| | 1.3278 | 1.5278 | 1.2437 | 0.2550 | 0.2342 | 1.0036 | 0.9990 | 1.5014 | 1.7543 | 1.3688 | 0.2426 | 0.6527 | 0.9935 | 0.9978 |
| | 1.6060 | 0.7940 | 0.9273 | 0.0893 | 0.5725 | 0.4859 | 0.9968 | 1.9359 | 0.6343 | 0.7477 | 0.3228 | 0.5112 | 0.7393 | 0.9858 |
| | 1.7816 | 1.9046 | 0.8581 | 0.0000 | 0.6167 | 0.2696 | 0.9978 | 1.5186 | 1.4606 | 1.2325 | 0.4080 | 0.3195 | 0.3463 | 0.9946 |
| | 1.5184 | 2.5236 | 1.1190 | 0.0492 | 0.5651 | 0.3686 | 0.7534 | 1.5478 | 0.7079 | 1.4157 | 0.0000 | 0.8126 | 0.1724 | 0.9933 |
| | 1.8570 | 1.5010 | 0.8963 | 0.8064 | 0.0058 | 0.7187 | 0.9902 | 1.7336 | 2.0598 | 1.2120 | 0.5092 | 0.0125 | 0.8722 | 0.9958 |
| | 1.5958 | 1.2107 | 0.9428 | 0.3270 | 0.3992 | 0.3206 | 0.9975 | 1.4296 | 0.2730 | 1.3836 | 0.1242 | 0.5903 | 0.5813 | 0.9965 |
| | 1.6734 | 1.0619 | 0.7851 | 0.2799 | 0.0000 | 0.9716 | 0.9688 | 1.5000 | 1.3676 | 1.2572 | 0.5541 | 1.1014 | 1.1311 | 0.9997 |
| | 1.1604 | 1.9508 | 1.1018 | 0.0650 | 0.5644 | 0.2925 | 0.9237 | 1.3508 | 1.4675 | 1.1974 | 0.4649 | 0.3763 | 0.4115 | 1.0000 |
| | 1.6986 | 1.3352 | 0.8006 | 0.0296 | 0.7747 | 0.9354 | 0.9946 | 1.4698 | 1.3164 | 0.8981 | 0.4092 | 0.5086 | 0.4819 | 0.9982 |
| | 1.4027 | 0.7382 | 0.9986 | 0.5288 | 0.4056 | 0.3857 | 0.9976 | 1.7235 | 1.6569 | 1.1175 | 0.1107 | 0.3020 | 0.5805 | 0.9998 |
| | 1.5304 | 1.5453 | 0.7991 | 0.6433 | 0.1884 | 1.3174 | 0.9944 | 1.9536 | 1.6732 | 1.1705 | 0.7501 | 0.0000 | 0.9957 | 0.8859 |
| | 1.2566 | 1.1253 | 1.2381 | 0.4390 | 0.0184 | 1.0886 | 0.9994 | 1.2708 | 1.4035 | 1.4106 | 0.2727 | 0.3360 | 0.8332 | 1.0000 |
| | 1.5509 | 3.3068 | 0.9944 | 0.0632 | 0.4647 | 0.8727 | 0.9887 | 1.5352 | 0.9224 | 1.2068 | 0.3707 | 0.4898 | 0.7060 | 0.9994 |
| | 1.4391 | 1.2676 | 1.1846 | 0.0001 | 0.4921 | 0.4924 | 0.8845 | 1.3840 | 1.2825 | 1.2662 | 0.5866 | 0.0085 | 0.7196 | 0.9794 |

| | | | | | | | | | | | | | | |
|---|---|---|---|---|---|---|---|---|---|---|---|---|---|---|
| | 1.4053 | 1.3198 | 0.9272 | 0.5144 | 0.1382 | 0.6841 | 0.9989 | 1.3967 | 1.5947 | 1.0311 | 0.2099 | 1.1680 | 0.4787 | 0.9637 |
| | 1.1730 | 0.3036 | 1.1874 | 0.0064 | 0.3102 | 0.9196 | 0.9997 | 1.6200 | 0.4221 | 1.0969 | 0.0000 | 0.5988 | 0.8356 | 0.9039 |
| | 1.7652 | 1.5903 | 0.8251 | 0.0016 | 0.6649 | 0.5732 | 0.9950 | 1.5872 | 0.9403 | 0.9393 | 0.0794 | 0.3680 | 0.5926 | 0.9963 |
| | 1.3442 | 2.1165 | 1.3871 | 0.1441 | 0.0000 | 0.7711 | 0.9947 | 1.3094 | 0.8591 | 1.4187 | 0.7344 | 0.5113 | 0.6106 | 0.9853 |
| | 1.5857 | 1.5549 | 1.1914 | 0.2305 | 0.2167 | 0.7281 | 0.9120 | 1.3859 | 1.8919 | 1.4493 | 0.0002 | 0.6994 | 1.0083 | 0.9940 |
| | 1.5814 | 1.8887 | 1.0967 | 0.2875 | 0.6210 | 0.0525 | 0.9998 | 1.4574 | 1.7180 | 1.3252 | 0.4700 | 0.6625 | 0.7918 | 0.9995 |
| | 1.5113 | 2.8309 | 1.1603 | 0.8892 | 1.0965 | 0.7276 | 0.9434 | 1.3056 | 0.6897 | 1.3472 | 0.4538 | 0.0062 | 0.3982 | 0.9998 |
| | 1.4939 | 0.9629 | 1.0658 | 0.0548 | 1.3439 | 0.3854 | 0.9994 | 1.6502 | 0.8322 | 1.1215 | 0.0000 | 1.1601 | 0.0003 | 0.9959 |
| | 1.6080 | 2.0066 | 0.8258 | 0.3963 | 0.8814 | 1.0707 | 0.9435 | 1.3035 | 1.2394 | 1.1889 | 0.3274 | 0.3589 | 1.0567 | 0.9859 |
| | 1.7672 | 1.4135 | 0.8667 | 0.1255 | 0.3962 | 0.4583 | 0.9972 | 1.6645 | 1.1391 | 0.9400 | 0.3301 | 0.6886 | 0.5891 | 0.9968 |
| | 1.6761 | 1.7846 | 0.9610 | 0.3455 | 0.7159 | 0.7581 | 0.9998 | 1.6453 | 1.4706 | 1.3076 | 0.3499 | 0.4877 | 0.2180 | 0.9806 |
| | - | - | - | - | - | - | - | 1.2446 | 0.9179 | 0.7909 | 0.5018 | 0.0879 | 0.5939 | 0.9997 |
| **Children – Social Condition** | | | | | | | | | | | | | | |
| tt11BI<br>tt12KC<br>tt13<br>tt13FJ<br>tt14FC<br>tt15<br>tt16<br>tt17<br>tt19<br>tt20<br>tt21UH<br>tt22HC | 1.5551 | 0.5508 | 0.8555 | 0.4129 | 0.2135 | 0.3186 | 0.8911 | 1.6307 | 1.1732 | 1.0700 | 0.0415 | 0.6399 | 0.3482 | 0.9945 |
| | 1.7794 | 2.5908 | 1.2193 | 0.4218 | 0.0000 | 1.1419 | 0.9891 | 1.5474 | 0.9305 | 1.1229 | 0.2558 | 0.5401 | 0.2280 | 0.9949 |
| | 2.1132 | 1.6003 | 0.6018 | 0.0260 | 0.8165 | 0.0001 | 0.9848 | 1.2361 | 2.2037 | 1.7490 | 0.5966 | 0.0878 | 0.7830 | 0.9997 |
| | 1.4140 | 1.1493 | 0.5420 | 0.0000 | 0.8964 | 0.4452 | 0.9913 | 1.3510 | 0.7407 | 0.5116 | 0.5085 | 0.3893 | 0.3022 | 0.9995 |
| | 1.6879 | 1.1032 | 0.7858 | 0.4436 | 0.0832 | 0.5853 | 0.9334 | 1.8712 | 1.6791 | 1.0815 | 0.8065 | 0.0000 | 0.9673 | 0.9757 |
| | 1.3634 | 1.0273 | 1.0306 | 0.0539 | 0.0621 | 0.5941 | 0.9995 | 1.5609 | 1.1085 | 1.1914 | 0.4394 | 0.0586 | 1.1675 | 0.9968 |
| | 1.6564 | 0.9938 | 0.5010 | 0.1956 | 0.5149 | 0.3920 | 0.9974 | 1.6105 | 0.5248 | 0.7753 | 0.4788 | 0.1607 | 0.5098 | 0.9997 |
| | 0.9940 | 2.1221 | 0.5153 | 0.2920 | 0.7661 | 0.1543 | 0.9919 | 0.8443 | 1.5655 | 0.6861 | 0.2662 | 0.3555 | 0.2401 | 0.9999 |

| | | | | | | | | | | | | | | |
|---|---|---|---|---|---|---|---|---|---|---|---|---|---|---|
| tt23<br>tt23MZ<br>tt24<br>tt24TS<br>tt25<br>tt26<br>tt26MH<br>tt28NM<br>tt3<br>tt4<br>tt43BC<br>tt45LA<br>tt46SE<br>tt48LM<br>tt49DM<br>tt5<br>tt50SA<br>tt53ED<br>tt54RA<br>tt55DC<br>tt56HM<br>tt57HA<br>tt58JR<br>tt59DN<br>tt60RG<br>tt62LL | 1.9430 | 2.3674 | 0.8042 | 0.9766 | 0.0133 | 1.1026 | 0.9747 | 1.7368 | 0.8347 | 0.8261 | 0.3643 | 0.1772 | 0.5070 | 0.9999 |
| | 1.2270 | 2.0231 | 0.6757 | 0.0882 | 0.0008 | 0.4949 | 0.8803 | 2.1118 | 1.2891 | 0.8352 | 0.3604 | 0.4145 | 0.2293 | 0.9972 |
| | 1.1420 | 1.5624 | 1.3812 | 0.0000 | 0.3652 | 0.8565 | 0.9826 | 1.5759 | 1.5387 | 1.1482 | 0.6642 | 0.2505 | 0.2649 | 0.9993 |
| | 1.6105 | 1.6221 | 0.8774 | 0.2872 | 0.4050 | 0.4817 | 0.9261 | 1.5629 | 1.2728 | 1.1063 | 0.1313 | 0.5953 | 0.9111 | 0.9990 |
| | 1.1197 | 0.8679 | 0.2899 | 0.0790 | 2.1814 | 0.0312 | 0.9858 | 0.6796 | 0.7015 | 0.5473 | 0.0931 | 0.4815 | 0.3756 | 0.9998 |
| | 1.5253 | 1.5746 | 0.7115 | 0.1611 | 0.5360 | 0.3370 | 0.9995 | 1.8095 | 0.9748 | 0.9494 | 0.5342 | 0.3044 | 0.5453 | 0.9859 |
| | 1.4172 | 1.4412 | 0.5102 | 0.2721 | 0.2982 | 0.6103 | 0.9998 | 1.9551 | 0.0100 | 0.8634 | 0.4078 | 0.2135 | 0.4641 | 0.9827 |
| | 1.5184 | 2.3034 | 0.5806 | 0.1489 | 0.7410 | 0.3326 | 0.9873 | 1.5329 | 2.0597 | 0.9134 | 0.1259 | 1.1105 | 0.2025 | 0.9799 |
| | 1.5953 | 1.9303 | 0.4271 | 0.4688 | 0.6906 | 0.2864 | 0.9826 | 1.0346 | 0.7166 | 0.8462 | 0.2183 | 0.4838 | 0.3656 | 0.9571 |
| | 1.5244 | 0.9959 | 1.0720 | 0.0000 | 0.6154 | 0.3373 | 0.9997 | 1.2155 | 1.8973 | 1.3861 | 0.1957 | 0.7486 | 0.5465 | 0.9998 |
| | 1.4147 | 1.0920 | 0.3420 | 0.3540 | 0.4516 | 0.3336 | 0.9956 | 1.5122 | 0.8852 | 0.9351 | 0.5804 | 0.7060 | 0.8661 | 0.9999 |
| | 1.2026 | 2.2100 | 0.2416 | 0.2683 | 0.3391 | 0.2691 | 0.9941 | 1.2188 | 1.0675 | 0.5925 | 0.5136 | 0.1130 | 0.5549 | 0.9992 |
| | 1.2236 | 1.8590 | 0.2648 | 0.2269 | 1.1429 | 0.0498 | 0.9931 | 1.2949 | 0.9973 | 0.6191 | 0.4573 | 0.5401 | 0.4598 | 0.9965 |
| | 1.5541 | 2.4117 | 0.2926 | 0.0000 | 0.9255 | 0.3375 | 0.9240 | 0.9102 | 1.0724 | 0.5238 | 0.1340 | 1.1178 | 0.1875 | 0.9991 |
| | 1.3465 | 1.1028 | 0.4245 | 0.2524 | 0.6064 | 0.1946 | 0.9901 | 1.4603 | 1.2807 | 0.7759 | 0.4410 | 0.3720 | 0.5326 | 0.9999 |
| | 1.5566 | 1.3399 | 0.4255 | 0.2298 | 0.9234 | 0.4536 | 0.9674 | 1.5918 | 1.3874 | 0.9844 | 0.0781 | 0.0100 | 1.0283 | 0.9980 |
| | 1.0267 | 1.0772 | 0.5251 | 0.0346 | 1.4862 | 0.2307 | 0.9910 | 1.2725 | 1.8090 | 0.5246 | 0.2277 | 0.6348 | 0.2178 | 0.9946 |
| | 1.0134 | 2.2024 | 0.5353 | 0.3314 | 0.4840 | 0.1288 | 0.9993 | 1.5844 | 1.4293 | 0.6742 | 0.2278 | 0.7158 | 0.2131 | 0.9937 |
| | 1.4133 | 1.5900 | 0.9857 | 0.4651 | 0.4513 | 0.5755 | 0.9850 | 1.4950 | 1.4836 | 1.0024 | 0.0812 | 0.4727 | 0.3857 | 0.9898 |
| | 1.6872 | 0.8906 | 1.0353 | 0.5773 | 0.3896 | 0.1923 | 0.9941 | 1.1392 | 1.1534 | 1.5771 | 0.0000 | 0.7912 | 0.9931 | 0.9877 |
| | 1.5983 | 1.6330 | 0.7475 | 0.2755 | 0.1962 | 0.3782 | 0.9971 | 1.5046 | 3.7674 | 1.3891 | 0.8704 | 2.0572 | 0.7276 | 0.9988 |

| | | | | | | | | | | | | | | |
|---|---|---|---|---|---|---|---|---|---|---|---|---|---|---|
| tt63DT | 1.3447 | 0.9604 | 0.6278 | 0.4737 | 0.3873 | 0.0560 | 1.0000 | 1.5627 | 2.0534 | 0.6061 | 0.0975 | 0.5562 | 0.4598 | 0.9494 |
| tt64GS | 1.4050 | 2.0746 | 0.7518 | 0.9924 | 0.9420 | 0.9722 | 0.9862 | 1.6099 | 1.3083 | 0.6638 | 0.2171 | 0.2639 | 0.8402 | 0.9502 |
| tt65JC | 1.2515 | 1.0385 | 1.1041 | 0.4060 | 0.0575 | 0.3243 | 0.9993 | 1.5907 | 1.3245 | 0.9733 | 0.1682 | 0.3611 | 0.3525 | 0.9984 |
| tt70BE | 1.7873 | 1.1981 | 0.4813 | 0.0000 | 0.8646 | 0.0007 | 0.9977 | 1.4895 | 1.3774 | 0.7030 | 0.1954 | 0.4396 | 0.5780 | 0.9990 |
| tt71MO | 1.4570 | 0.8851 | 0.8733 | 0.3481 | 0.4115 | 0.4917 | 0.9989 | 1.5330 | 1.4891 | 1.4232 | 0.3338 | 0.6317 | 1.0583 | 0.9600 |
| tt72BH | 1.5005 | 0.6817 | 0.5404 | 0.2040 | 1.1931 | 0.3459 | 0.9961 | 1.7665 | 0.8631 | 0.7009 | 0.4277 | 0.4726 | 0.2756 | 0.9979 |
| tt73CM | 1.3740 | -0.192 | 0.3160 | 0.0008 | 0.2103 | 0.4557 | 0.9971 | 1.4156 | 0.3066 | 0.5608 | 0.5642 | 0.5089 | 0.5432 | 0.9997 |
| tt78IA | 1.3333 | 1.1915 | 0.3394 | 0.4207 | 1.7374 | 0.0203 | 0.9559 | 1.9083 | -0.138 | 0.2912 | 0.2938 | 0.5149 | 0.2147 | 0.9799 |
| tt79DA | 1.6265 | 1.2663 | 0.5805 | 0.3110 | 0.1578 | 0.3699 | 0.9998 | 1.6074 | 0.2194 | 0.7936 | 0.6912 | 0.1130 | 0.6774 | 0.9915 |
| tt83PD | 1.2390 | 1.9152 | 0.3802 | 0.2670 | 0.4493 | 0.3937 | 0.9972 | 1.6995 | 0.8471 | 0.9238 | 0.4019 | 0.0195 | 0.4203 | 0.9980 |
| tt84SA | 1.4658 | 0.5428 | 0.3461 | 0.3503 | 0.9383 | 0.6922 | 0.9597 | 1.6817 | 0.9222 | 0.8796 | 0.7106 | 0.5652 | 1.2636 | 0.9973 |
| tt8BB | 1.1857 | 0.9707 | 0.4544 | 0.0663 | 0.7334 | 0.3580 | 0.9998 | 1.0792 | 0.8892 | 0.9067 | 0.8970 | 0.3389 | 0.7722 | 0.9829 |
| | 1.4825 | 1.3231 | 0.6024 | 0.4206 | 0.3596 | 0.2581 | 0.9994 | 1.5632 | 1.0192 | 0.9207 | 0.4000 | 0.3153 | 0.3368 | 0.9972 |
| | 1.2278 | 2.1253 | 0.4747 | 0.0669 | 0.8666 | 0.3741 | 0.9994 | 0.9410 | 1.4868 | 0.9434 | 0.0583 | 0.5774 | 0.4180 | 0.9994 |
| | 1.8193 | 1.9295 | 0.6849 | 0.2287 | 0.4647 | 0.5928 | 0.9904 | 1.3099 | 0.6818 | 1.3770 | 0.0639 | 0.2386 | 1.0082 | 0.9905 |
| | 0.7900 | 1.0583 | 0.5047 | 0.2714 | 0.4420 | 0.2175 | 0.9996 | 1.1089 | -0.317 | 0.6565 | 0.1151 | 0.6589 | 0.2076 | 0.9470 |
| | 1.7764 | 2.1089 | 0.9019 | 0.4374 | 0.3786 | 1.0027 | 0.9880 | 1.5422 | 0.9361 | 1.2582 | 0.2749 | 0.2637 | 0.6778 | 0.9753 |
| | 1.9981 | 1.3013 | 0.4940 | 0.3361 | 0.4394 | 0.2698 | 0.9806 | 1.6391 | 0.6296 | 1.0305 | 0.2568 | 0.5401 | 0.2325 | 0.9323 |
| | 1.4670 | 1.6673 | 0.8227 | 0.5225 | 0.5073 | 0.5326 | 0.9933 | 1.2572 | 0.9423 | 1.5799 | 0.6733 | 0.1823 | 0.5973 | 0.9999 |
| | 1.1843 | 1.3220 | 0.3489 | 0.0465 | 0.7364 | 0.2784 | 0.9987 | 1.1818 | 0.3669 | 0.4972 | 0.2238 | 0.9325 | 0.5107 | 0.9993 |
| | 1.7042 | 1.0494 | 0.8032 | 0.5049 | 0.2637 | 0.4999 | 0.9208 | 1.5995 | 0.9899 | 0.8891 | 0.4653 | 0.3893 | 0.4764 | 0.9733 |

<table>
<tr><th colspan="15">Adults – Alone Condition</th></tr>
<tr><td rowspan="20">tts10<br>tts101<br>tts102<br>tts103<br>tts106<br>tts113<br>tts114<br>tts121<br>tts122<br>tts123<br>tts124<br>tts125<br>tts126<br>tts127<br>tts128<br>tts129<br>tts132<br>tts133<br>tts135<br>tts140<br>tts145<br>tts146<br>tts147<br>tts15<br>tts18<br>tts21<br>tts22<br>tts23<br>tts24<br>tts25<br>tts26</td><td>0.7606</td><td>2.7171</td><td>0.9965</td><td>0.0000</td><td>1.5818</td><td>0.0340</td><td>0.7204</td><td>1.2694</td><td>3.2513</td><td>1.0140</td><td>0.4221</td><td>0.2479</td><td>0.0834</td><td>0.9997</td></tr>
<tr><td>1.9523</td><td>3.3935</td><td>0.2076</td><td>0.0503</td><td>1.0242</td><td>0.0046</td><td>0.9055</td><td>2.0331</td><td>2.6841</td><td>0.3564</td><td>0.0475</td><td>0.8254</td><td>0.0026</td><td>0.9940</td></tr>
<tr><td>1.4999</td><td>0.9556</td><td>0.2912</td><td>0.3781</td><td>1.1622</td><td>0.0182</td><td>0.9998</td><td>1.9046</td><td>1.4642</td><td>0.4341</td><td>0.5006</td><td>0.2418</td><td>0.4161</td><td>0.8781</td></tr>
<tr><td>1.5175</td><td>2.3165</td><td>0.3162</td><td>0.1406</td><td>0.5095</td><td>0.2682</td><td>0.9788</td><td>1.5459</td><td>3.1242</td><td>0.4310</td><td>0.7111</td><td>0.0000</td><td>0.0525</td><td>0.9998</td></tr>
<tr><td>1.5281</td><td>2.4429</td><td>1.1449</td><td>0.4663</td><td>0.3642</td><td>0.4689</td><td>0.8019</td><td>2.0383</td><td>2.7734</td><td>1.1997</td><td>0.0242</td><td>0.0000</td><td>0.4991</td><td>0.9518</td></tr>
<tr><td>0.8774</td><td>2.7859</td><td>1.0000</td><td>0.0839</td><td>0.4166</td><td>0.3236</td><td>0.7315</td><td>1.1931</td><td>2.1094</td><td>1.0064</td><td>0.2546</td><td>0.8885</td><td>0.1203</td><td>0.9977</td></tr>
<tr><td>1.2961</td><td>2.0213</td><td>1.0587</td><td>0.1989</td><td>0.4898</td><td>0.2505</td><td>0.9845</td><td>1.4022</td><td>2.3299</td><td>1.0883</td><td>0.0780</td><td>0.6145</td><td>0.2140</td><td>0.9998</td></tr>
<tr><td>1.4933</td><td>2.1080</td><td>0.4129</td><td>0.3296</td><td>0.1916</td><td>0.3993</td><td>0.9978</td><td>1.7501</td><td>2.0673</td><td>0.3971</td><td>0.2407</td><td>0.6405</td><td>0.1160</td><td>0.9998</td></tr>
<tr><td>1.2073</td><td>1.7181</td><td>0.7653</td><td>0.0238</td><td>1.0174</td><td>0.6935</td><td>0.6627</td><td>1.9525</td><td>1.9202</td><td>0.4439</td><td>0.4982</td><td>0.3265</td><td>0.1125</td><td>0.9087</td></tr>
<tr><td>1.3235</td><td>1.5331</td><td>0.3609</td><td>0.2283</td><td>0.6405</td><td>0.3757</td><td>1.0000</td><td>1.6856</td><td>2.0565</td><td>0.3861</td><td>0.2777</td><td>0.5149</td><td>0.1875</td><td>0.9834</td></tr>
<tr><td>1.1408</td><td>1.3812</td><td>0.2365</td><td>0.1983</td><td>0.7453</td><td>0.0340</td><td>0.9931</td><td>1.1835</td><td>2.0307</td><td>0.4135</td><td>0.0567</td><td>0.7159</td><td>0.2244</td><td>0.9992</td></tr>
<tr><td>1.1939</td><td>0.7079</td><td>0.9873</td><td>0.2206</td><td>0.5007</td><td>0.2629</td><td>0.9939</td><td>1.7695</td><td>1.9231</td><td>0.9271</td><td>0.3039</td><td>0.0487</td><td>0.3118</td><td>0.9760</td></tr>
<tr><td>1.4970</td><td>2.0750</td><td>0.6096</td><td>0.4682</td><td>0.2008</td><td>0.4117</td><td>0.9991</td><td>2.4499</td><td>2.9435</td><td>0.5148</td><td>0.0974</td><td>1.0437</td><td>0.3433</td><td>0.9998</td></tr>
<tr><td>1.3686</td><td>1.7832</td><td>0.4146</td><td>0.0552</td><td>0.7912</td><td>0.6415</td><td>0.7539</td><td>1.5492</td><td>1.5107</td><td>0.7662</td><td>0.2407</td><td>0.6125</td><td>0.3859</td><td>0.9974</td></tr>
<tr><td>1.6660</td><td>2.3028</td><td>0.9425</td><td>0.2929</td><td>0.8167</td><td>0.0042</td><td>0.9711</td><td>1.7426</td><td>2.0112</td><td>1.0011</td><td>0.5747</td><td>0.0935</td><td>0.2475</td><td>0.9068</td></tr>
<tr><td>1.1408</td><td>2.6806</td><td>0.2662</td><td>0.5734</td><td>0.5860</td><td>0.0375</td><td>0.9029</td><td>1.4796</td><td>1.7528</td><td>0.4218</td><td>0.0866</td><td>0.7376</td><td>0.0216</td><td>0.9985</td></tr>
<tr><td>1.2851</td><td>1.7385</td><td>0.2507</td><td>0.2467</td><td>0.6039</td><td>0.0872</td><td>0.9993</td><td>1.5449</td><td>2.8694</td><td>0.3770</td><td>0.0004</td><td>1.0806</td><td>0.1918</td><td>0.9959</td></tr>
<tr><td>1.0972</td><td>2.0623</td><td>0.5533</td><td>0.0274</td><td>0.6405</td><td>0.0889</td><td>0.8902</td><td>1.8777</td><td>2.4090</td><td>0.5367</td><td>0.0000</td><td>0.8720</td><td>0.0319</td><td>0.9645</td></tr>
<tr><td>1.2907</td><td>1.8497</td><td>1.0871</td><td>0.1407</td><td>0.7933</td><td>0.4252</td><td>0.9626</td><td>1.5047</td><td>2.0448</td><td>1.1725</td><td>0.7100</td><td>0.0070</td><td>0.3519</td><td>0.9728</td></tr>
<tr><td>1.9351</td><td>3.5461</td><td>1.1708</td><td>0.0007</td><td>0.4357</td><td>0.8865</td><td>0.8865</td><td>1.4274</td><td>2.3032</td><td>1.1585</td><td>0.5576</td><td>0.4569</td><td>0.5509</td><td>0.9992</td></tr>
</table>

| | | | | | | | | | | | | | | |
|---|---|---|---|---|---|---|---|---|---|---|---|---|---|---|
| tts29<br>tts3<br>tts30<br>tts31<br>tts32<br>tts33<br>tts36<br>tts37<br>tts38<br>tts41<br>tts44<br>tts45<br>tts6<br>tts7<br>tts8<br>tts9<br>tts134 | 1.1495 | 1.6409 | 0.3702 | 0.2590 | 0.6154 | 0.3275 | 0.9955 | 1.3380 | 3.4557 | 0.3702 | 0.0139 | 0.4332 | 0.1233 | 0.9989 |
| | 1.3935 | 2.4299 | 1.2352 | 0.2423 | 0.3316 | 0.3825 | 0.8993 | 1.5007 | 2.4036 | 1.3067 | 0.1894 | 0.0044 | 0.4922 | 0.8959 |
| | 1.4606 | 1.6013 | 0.8526 | 0.3138 | 0.8774 | 0.4291 | 0.9771 | 1.6446 | 2.9303 | 1.0217 | 0.1430 | 0.6153 | 0.6533 | 0.9996 |
| | 1.3680 | 1.3237 | 0.2491 | 0.0669 | 0.8124 | 0.0028 | 0.9206 | 1.2057 | 0.9173 | 0.4186 | 0.2854 | 0.5377 | 0.3284 | 0.9998 |
| | 1.9751 | 2.1161 | 0.6646 | 0.0598 | 0.2167 | 0.8827 | 0.9592 | 1.4058 | 0.8194 | 0.9757 | 0.2157 | 0.8164 | 0.4690 | 0.9846 |
| | 1.8132 | 3.3350 | 0.8453 | 0.1452 | 1.5124 | 0.5176 | 0.1133 | 1.5519 | 2.0323 | 1.0362 | 0.3184 | 0.4144 | 0.4049 | 0.9951 |
| | 1.2424 | 1.6619 | 0.3506 | 0.3573 | 0.4306 | 0.2218 | 0.9998 | 1.5127 | 2.7188 | 0.3621 | 0.2314 | 0.2387 | 0.2084 | 0.9965 |
| | 0.9644 | 2.3377 | 0.3799 | 0.0676 | 1.1931 | 0.1553 | 1.0000 | 1.4276 | 2.7842 | 0.4642 | 0.0000 | 0.0000 | 0.3975 | 0.9124 |
| | 1.5714 | 1.3510 | 0.5440 | 0.2941 | 0.5015 | 0.5043 | 0.9992 | 1.7276 | 1.3963 | 0.5150 | 0.4260 | 0.3409 | 0.2573 | 0.9093 |
| | 1.1057 | 0.9649 | 0.4779 | 0.1951 | 0.7080 | 0.2203 | 0.9991 | 1.5092 | 2.1398 | 0.4476 | 0.0299 | 0.8917 | 0.1250 | 0.9929 |
| | 1.2519 | 2.5695 | 0.9835 | 0.1266 | 0.8436 | 0.0880 | 0.9854 | 1.5426 | 3.2605 | 0.9627 | 0.0144 | 1.0469 | 0.1275 | 0.9997 |
| | 1.7025 | 1.7781 | 0.7033 | 0.6243 | 0.1375 | 1.0090 | 0.9802 | 1.1053 | 1.2206 | 1.2078 | 0.4841 | 0.8418 | 0.9411 | 0.9966 |
| | 1.1026 | 1.8731 | 0.3894 | 0.0000 | 0.8501 | 0.1459 | 0.9767 | 1.5245 | 2.4675 | 0.5131 | 0.2177 | 1.0424 | 0.1829 | 0.9994 |
| | 1.0620 | 2.2020 | 0.4000 | 0.2754 | 0.3861 | 0.2401 | 0.9968 | 1.5664 | 1.3087 | 0.4809 | 0.4995 | 0.4145 | 0.0679 | 0.9993 |
| | 1.0326 | 2.0527 | 0.9999 | 0.0731 | 1.0868 | 0.0375 | 0.6886 | 1.3566 | 4.3481 | 1.0091 | 0.0983 | 0.8415 | 0.1582 | 0.9991 |
| | 1.6128 | 1.6197 | 0.5619 | 0.3803 | 0.6959 | 0.1314 | 0.9386 | 1.4684 | 2.1182 | 0.8872 | 0.0963 | 0.0879 | 0.7589 | 0.9433 |
| | 0.9663 | 1.9516 | 0.9482 | 0.3316 | 0.5086 | 0.3538 | 0.1315 | 1.5431 | 2.0770 | 0.8910 | 0.5561 | 0.5976 | 0.4074 | 0.8609 |
| | 1.1973 | 1.3445 | 0.9428 | 0.2462 | 0.9472 | 0.4165 | 0.9856 | 1.3405 | 1.8314 | 0.8881 | 0.2971 | 0.0025 | 0.6510 | 0.9984 |
| | 0.9790 | 2.3196 | 0.3269 | 0.0853 | 1.5197 | 0.0236 | 0.9668 | 1.2748 | 2.4795 | 0.4667 | 0.3572 | 0.6669 | 0.0744 | 0.9695 |
| | 1.6037 | 1.1123 | 0.5575 | 0.9178 | 0.0906 | 0.4987 | 0.5504 | 1.3460 | 2.2129 | 0.7226 | 0.1198 | 1.0841 | 0.4073 | 0.9742 |
| | 1.7405 | 2.8458 | 0.2260 | 0.1138 | 0.8907 | 0.0686 | 0.9985 | 1.1635 | 3.0078 | 0.3932 | 0.3520 | 0.3242 | 0.1484 | 0.9984 |

| | | | | | | | | | | | | | | |
|---|---|---|---|---|---|---|---|---|---|---|---|---|---|---|
| | 1.2183 | 1.5342 | 1.0433 | 0.1251 | 0.7325 | 0.4017 | 0.9987 | 1.3298 | 1.1375 | 1.0530 | 0.0000 | 0.7159 | 0.3809 | 0.9302 |
| | 0.9664 | 2.0323 | 0.9968 | 0.2214 | 0.7625 | 0.3303 | 0.4454 | 1.2604 | 2.7697 | 1.0663 | 0.2063 | 0.8470 | 0.2925 | 0.9998 |
| | 1.7862 | 1.7430 | 0.6757 | 0.4447 | 0.0285 | 0.9638 | 0.8508 | 1.5102 | 0.0292 | 0.8170 | 0.0000 | 2.4993 | 0.9042 | 0.9526 |
| | 1.1859 | 1.8736 | 0.2966 | 0.2468 | 0.6381 | 0.1575 | 0.9763 | 1.2534 | 1.6377 | 0.4575 | 0.0658 | 0.8377 | 0.1208 | 0.9975 |
| | 1.3315 | 2.0326 | 0.5115 | 0.0979 | 0.3771 | 0.4183 | 0.9968 | 1.9474 | 2.8138 | 0.5619 | 0.5047 | 0.0160 | 0.5099 | 0.9947 |
| | 1.0876 | 1.8208 | 1.0076 | 0.2727 | 0.1361 | 0.1425 | 0.9567 | 1.7125 | 1.9486 | 0.9105 | 0.3161 | 0.4980 | 0.2102 | 0.9555 |
| | - | - | - | - | - | - | - | 1.9021 | 3.0046 | 1.2503 | 0.5975 | 0.0000 | 0.4307 | 0.9796 |
| **Adults – Social Condition** | | | | | | | | | | | | | | |
| tts1<br>tts104<br>tts105<br>tts108<br>tts109<br>tts11<br>tts110<br>tts111<br>tts112<br>tts115<br>tts116<br>tts117<br>tts119<br>tts12<br>tts120<br>tts13<br>tts130<br>tts131<br>tts136<br>tts137<br>tts138 | 1.2671 | 3.4369 | 0.2595 | 0.2304 | 0.4647 | 0.1619 | 0.9975 | 1.2008 | 2.1042 | 0.4174 | 0.2527 | 0.4844 | 0.1674 | 0.9824 |
| | 0.9306 | 1.9425 | 0.2809 | 0.2442 | 0.5548 | 0.1125 | 0.9195 | 1.2005 | 2.4790 | 0.3771 | 0.4115 | 0.6757 | 0.0438 | 0.9809 |
| | 1.2962 | 2.9207 | 0.9393 | 0.0549 | 1.6802 | 0.0001 | 0.3261 | 1.2943 | 2.2292 | 0.9545 | 0.2821 | 0.7633 | 0.0675 | 0.9937 |
| | 1.6937 | 1.5086 | 0.4556 | 0.6523 | 0.4036 | 0.9113 | 0.2345 | 2.0129 | 1.9274 | 0.5113 | 0.6697 | 0.0220 | 0.3705 | 0.8873 |
| | 1.0944 | 1.9838 | 0.9334 | 0.2881 | 0.6698 | 0.1725 | 0.9962 | 1.1268 | 2.7941 | 0.9472 | 0.4527 | 0.6907 | 0.1711 | 0.9384 |
| | 2.0387 | 1.5353 | 0.5213 | 0.9017 | 0.1722 | 0.8692 | 0.0255 | 1.9619 | 2.3864 | 0.8041 | 0.0000 | 0.1031 | 1.1054 | 0.4234 |
| | 1.1514 | 2.4552 | 1.1557 | 0.4521 | 0.5451 | 0.3597 | 0.9989 | 1.3035 | 2.2509 | 1.2122 | 0.0620 | 0.3091 | 0.4069 | 0.9824 |
| | 1.0426 | 1.7393 | 1.1065 | 0.0860 | 0.4396 | 0.2906 | 0.9987 | 1.0913 | 1.9552 | 1.0908 | 0.2771 | 0.5047 | 0.2583 | 0.9814 |
| | 1.4320 | 1.3247 | 0.4703 | 0.0735 | 1.1337 | 0.2217 | 0.9816 | 1.4200 | 1.9621 | 0.6432 | 0.3382 | 0.1631 | 0.4467 | 0.9857 |
| | 1.1146 | 1.9512 | 1.1059 | 0.0807 | 0.1628 | 0.5532 | 0.9792 | 1.2540 | 1.7659 | 1.1252 | 0.4275 | 0.0661 | 0.4830 | 0.9943 |
| | 1.0904 | 3.0478 | 1.1849 | 0.0156 | 0.5401 | 0.3486 | 0.9921 | 1.1892 | 1.9126 | 1.1444 | 0.0000 | 0.1871 | 0.4919 | 0.9503 |
| | 1.4099 | 1.7356 | 0.5435 | 0.7284 | 0.0000 | 0.7045 | 0.9552 | 1.8923 | 2.2178 | 0.5663 | 0.6056 | 0.0001 | 0.6334 | 0.9876 |
| | 1.2149 | 1.4065 | 0.2884 | 0.2125 | 0.6656 | 0.2122 | 1.0000 | 1.6313 | 1.7610 | 0.5266 | 0.4459 | 0.1633 | 0.4951 | 0.9825 |

| | | | | | | | | | | | | | | |
|---|---|---|---|---|---|---|---|---|---|---|---|---|---|---|
| tts139<br>tts14<br>tts141<br>tts142<br>tts143<br>tts144<br>tts150<br>tts152<br>tts153<br>tts154<br>tts155<br>tts16<br>tts17<br>tts19<br>tts2<br>tts20<br>tts27<br>tts28<br>tts34<br>tts35<br>tts39<br>tts4<br>tts40<br>tts42<br>tts43<br>tts46<br>tts47<br>tts5 | 1.1064 | 2.8829 | 0.2739 | 0.2787 | 0.5705 | 0.1425 | 0.6357 | 0.9780 | 2.6728 | 0.4090 | 0.3418 | 0.4360 | 0.2025 | 0.9991 |
| | 0.8893 | 1.5817 | 0.4571 | 0.0520 | 0.2889 | 0.4105 | 0.9997 | 1.1226 | 2.0071 | 0.4522 | 0.1270 | 1.1343 | 0.1875 | 0.9988 |
| | 1.6923 | 2.1404 | 0.6502 | 0.3714 | 1.6591 | 0.7325 | 0.9693 | 1.8429 | 2.1318 | 0.6851 | 0.0000 | 0.2093 | 0.6542 | 0.9999 |
| | 1.5091 | 1.4613 | 0.3890 | 0.1645 | 0.6977 | 0.3041 | 0.9969 | 1.5376 | 1.1300 | 0.6871 | 0.4699 | 0.2889 | 0.8360 | 0.9450 |
| | 1.4178 | 2.7326 | 0.1998 | 0.0002 | 1.1616 | 0.1575 | 0.9963 | 1.4818 | 2.3224 | 0.3986 | 0.0000 | 0.6089 | 0.2374 | 0.9987 |
| | 1.0205 | 1.6745 | 0.3419 | 0.3287 | 0.3893 | 0.2873 | 0.9981 | 1.3363 | 1.8066 | 0.5321 | 0.1149 | 0.5652 | 0.4583 | 0.9954 |
| | 1.3062 | 2.3420 | 1.0083 | 0.2980 | 0.3014 | 0.5478 | 0.9410 | 1.8677 | 2.5453 | 0.9828 | 0.7246 | 0.0630 | 0.3525 | 0.9695 |
| | 1.4965 | 1.6106 | 0.4091 | 0.2160 | 0.5903 | 0.2969 | 0.9999 | 1.4499 | 1.9641 | 0.6457 | 0.0880 | 0.5559 | 0.3253 | 0.9994 |
| | 1.3077 | 2.9679 | 0.2351 | 0.0813 | 1.3345 | 0.0975 | 0.9888 | 1.6944 | 1.5356 | 0.3957 | 0.5064 | 0.2859 | 0.0525 | 0.8933 |
| | 1.5020 | 1.4331 | 0.3427 | 0.3811 | 0.3989 | 0.4073 | 0.8484 | 2.4910 | 3.5551 | 0.5416 | 0.6716 | 0.1130 | 0.6325 | 0.9594 |
| | 1.5458 | 1.3281 | 0.4019 | 0.2880 | 0.4898 | 0.3096 | 0.9977 | 1.7397 | 1.8016 | 0.5966 | 0.3543 | 0.1383 | 0.5187 | 0.9981 |
| | 1.2393 | 0.8996 | 0.6514 | 0.0164 | 0.3953 | 0.7925 | 0.9815 | 1.4899 | 1.9079 | 0.7018 | 0.6080 | 0.0659 | 0.8826 | 0.9992 |
| | 1.9105 | 1.9021 | 0.2425 | 0.0799 | 0.7454 | 0.0526 | 0.9782 | 1.7532 | 2.2206 | 0.3913 | 0.0756 | 0.7088 | 0.1403 | 0.9988 |
| | 1.1996 | 2.0277 | 0.6749 | 0.3876 | 0.9922 | 0.6992 | 0.8243 | 1.6537 | 3.0962 | 0.6345 | 0.1775 | 0.0346 | 0.6386 | 0.7270 |
| | 0.9951 | 2.7041 | 0.2831 | 0.2192 | 0.6073 | 0.1125 | 0.9986 | 1.1347 | 1.3100 | 0.4505 | 0.3316 | 0.4898 | 0.1495 | 0.9915 |
| | 0.7906 | 2.9923 | 1.1112 | 0.4382 | 0.5401 | 0.3721 | 0.2975 | 0.9273 | 2.6408 | 1.2051 | 0.0976 | 0.5491 | 0.2175 | 0.6753 |
| | 2.0155 | 1.8291 | 0.4435 | 0.7287 | 0.0879 | 0.7136 | 0.9507 | 1.9028 | 1.5573 | 0.5470 | 0.7214 | 0.0635 | 0.4570 | 0.9102 |
| | 1.3030 | 1.7506 | 0.3314 | 0.0000 | 1.2423 | 0.2223 | 0.9890 | 1.4240 | 2.4660 | 0.4295 | 0.2798 | 0.0310 | 0.3419 | 0.9740 |
| | 1.9632 | 1.4600 | 0.2356 | 0.2955 | 0.3372 | 0.1001 | 0.8587 | 2.0207 | 1.4729 | 0.2717 | 0.1054 | 0.3893 | 0.2144 | 0.9386 |
| | 1.1652 | 1.6067 | 0.9299 | 0.2494 | 0.9420 | 0.1396 | 0.8892 | 1.6188 | 2.0663 | 0.9742 | 0.4043 | 0.0957 | 0.0975 | 0.9971 |
| | 1.3177 | 1.8047 | 0.3588 | 0.1526 | 0.7410 | 0.3140 | 0.9991 | 1.6538 | 1.9946 | 0.4977 | 0.3939 | 0.0879 | 0.2648 | 0.9997 |

| | | | | | | | | | | | | | | |
|---|---|---|---|---|---|---|---|---|---|---|---|---|---|---|
| | 1.3102 | 1.7892 | 0.3472 | 0.3639 | 0.5578 | 0.1432 | 0.9865 | 1.3641 | 1.9383 | 0.4612 | 0.3349 | 0.3707 | 0.1162 | 0.9657 |
| | 1.3095 | 2.5318 | 0.1693 | 0.0025 | 0.8461 | 0.0023 | 0.9110 | 1.4900 | 2.8550 | 0.3635 | 0.0553 | 1.8362 | 0.1192 | 0.9830 |
| | 1.2766 | 2.5174 | 0.3430 | 0.3370 | 0.5652 | 0.1261 | 0.9991 | 1.7026 | 2.3425 | 0.3460 | 0.3326 | 0.4973 | 0.1153 | 0.9997 |
| | 1.0803 | 1.0641 | 0.5738 | 0.2169 | 0.1382 | 0.4259 | 0.9969 | 1.3753 | 1.9834 | 0.5291 | 0.2161 | 0.7410 | 0.2130 | 0.9933 |
| | 1.0985 | 2.4418 | 0.2443 | 0.3053 | 0.8969 | 0.0380 | 0.9581 | 1.0489 | 2.6792 | 0.3838 | 0.3873 | 0.4396 | 0.1109 | 0.9918 |
| | 0.9453 | 2.3051 | 0.9061 | 0.4510 | 0.3642 | 0.2301 | 0.8468 | 1.1926 | 0.9056 | 0.9074 | 0.0080 | 0.7725 | 0.2705 | 0.9982 |
| | 1.4058 | 2.1765 | 0.3665 | 0.1373 | 0.5775 | 0.3706 | 1.0000 | 1.4419 | 1.6566 | 0.4638 | 0.0001 | 1.7439 | 0.1873 | 0.9886 |
| | 1.4635 | 2.6232 | 0.1806 | 0.1336 | 0.8600 | 0.0506 | 0.6468 | 1.3762 | 2.0872 | 0.3969 | 0.2701 | 0.5648 | 0.1973 | 0.9969 |
| | 1.0632 | 2.4395 | 0.3831 | 0.3631 | 0.5330 | 0.3278 | 0.9880 | 1.4449 | 1.2449 | 0.4494 | 0.1650 | 0.5401 | 0.1570 | 0.9993 |
| | 1.1574 | 1.4436 | 0.2591 | 0.3913 | 0.4108 | 0.1434 | 0.9783 | 1.4418 | 2.1422 | 0.3292 | 0.3412 | 0.5596 | 0.0001 | 0.9725 |
| | 1.2001 | 1.6897 | 1.0636 | 0.4500 | 0.2886 | 0.3504 | 0.9685 | 1.2586 | 1.6197 | 1.0602 | 0.2087 | 0.5043 | 0.3581 | 0.9891 |
| | 1.3045 | 1.0803 | 0.6066 | 0.4150 | 0.2703 | 0.5187 | 0.9997 | 0.8247 | 0.6071 | 1.3879 | 0.0000 | 0.7158 | 0.6809 | 0.9520 |
| | 1.2803 | 0.9875 | 0.2894 | 0.2384 | 0.7410 | 0.3021 | 0.9610 | 1.5683 | 1.5665 | 0.7378 | 0.4377 | 0.5806 | 0.8632 | 0.8617 |
| | 1.5847 | 1.3060 | 0.4623 | 0.4588 | 0.6219 | 0.0090 | 0.9882 | 1.2975 | 0.7292 | 0.9319 | 0.1138 | 0.1860 | 0.7089 | 0.8912 |
| | 1.2616 | 2.2123 | 1.0086 | 0.4870 | 0.1539 | 0.5815 | 0.9910 | 1.5651 | 2.3471 | 1.0009 | 0.6686 | 0.1238 | 0.8608 | 0.9937 |

**Figure S2: Individual estimated Diffusion model distributions.** Individual RT distributions (correct trials, black curve) in children (A) and adults (B) for each trial type (Numerosity or Phonological comparison) and each condition (Social/Alone) and estimated distribution according to the Diffusion model estimated parameters (red curve).

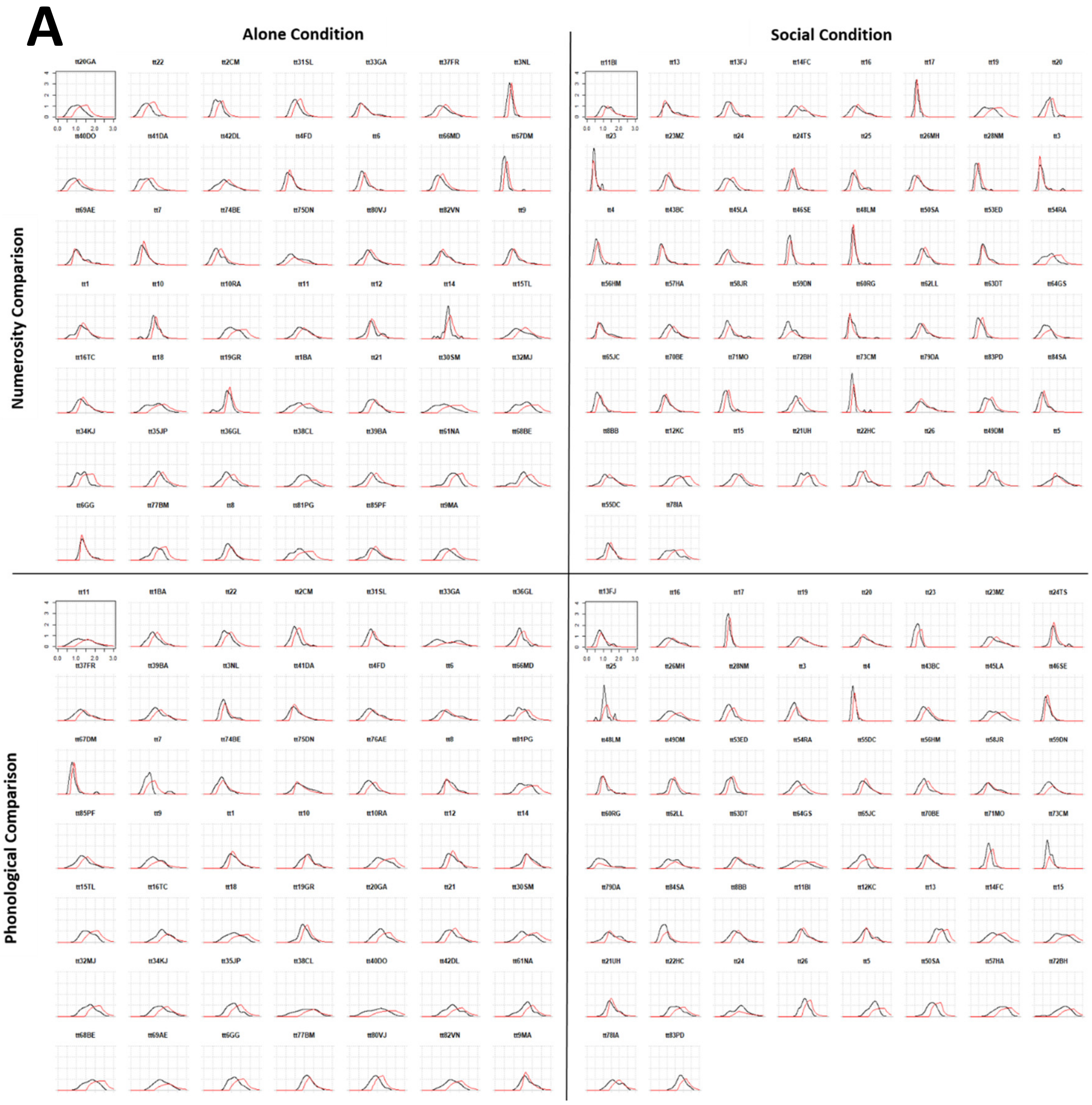

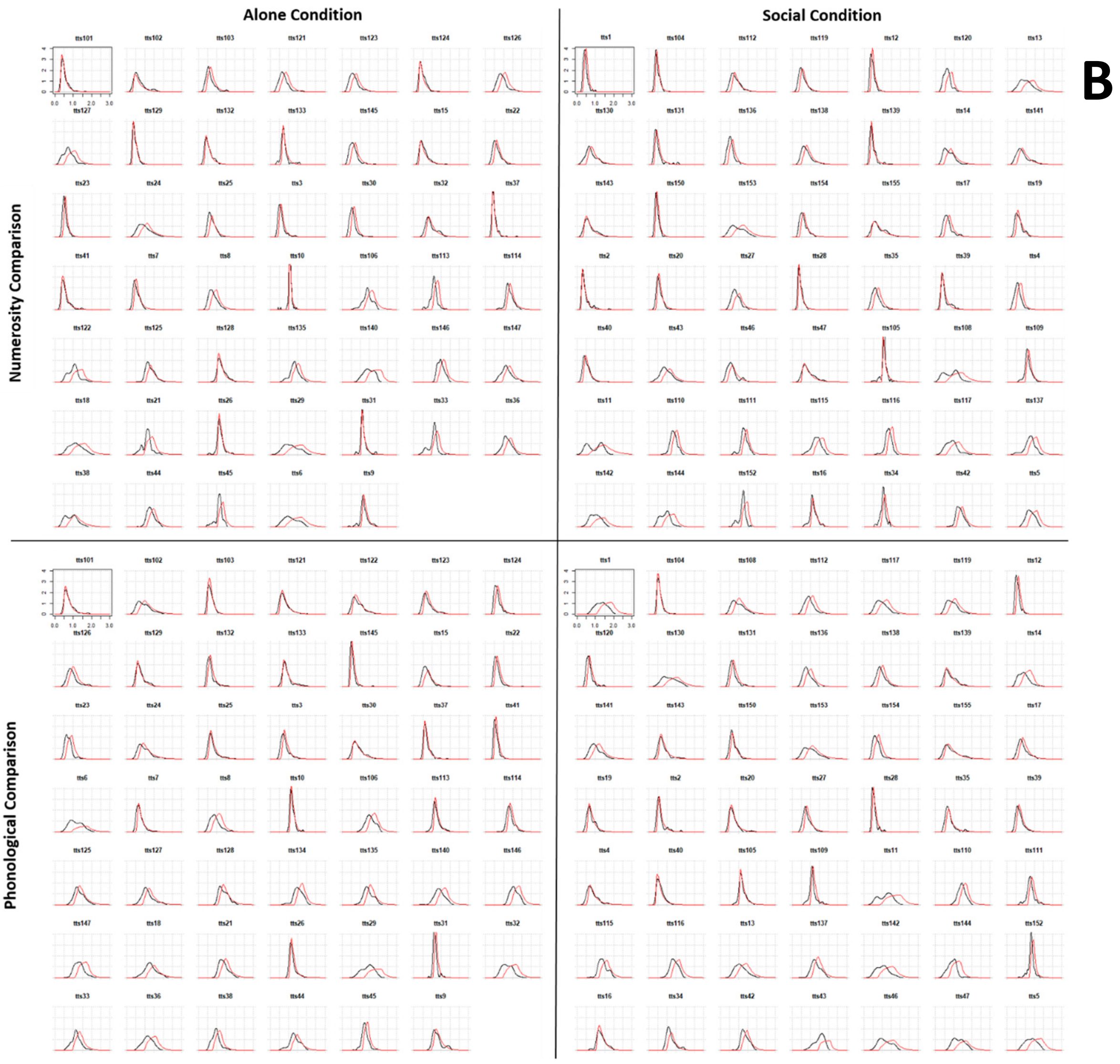
Alone Condition
Social Condition
B
Numerosity Comparison
Phonological Comparison
